# Nuclear softening mediated by Sun2 suppression delays mechanical stress-induced cellular senescence

**DOI:** 10.1038/s41420-023-01467-1

**Published:** 2023-05-17

**Authors:** Xianlin Yue, Jie Cui, Zewei Sun, Lei Liu, Ying Li, Liwei Shao, Qi Feng, Ziyue Wang, William S. Hambright, Yan Cui, Johnny Huard, Yanling Mu, Xiaodong Mu

**Affiliations:** 1 Shandong First Medical University & Shandong Academy of Medical Sciences, Jinan, Shandong China; 2 Steadman Philippon Research Institute, Center for Regenerative Sports Medicine, Vail, CO USA; 3 Department of Orthopaedic Surgery, McGovern Medical School, University of Texas Health Science Center at Houston, Houston, TX USA

**Keywords:** Senescence, Nuclear envelope

## Abstract

Nuclear decoupling and softening are the main cellular mechanisms to resist mechanical stress-induced nuclear/DNA damage, however, its molecular mechanisms remain much unknown. Our recent study of Hutchinson-Gilford progeria syndrome (HGPS) disease revealed the role of nuclear membrane protein Sun2 in mediating nuclear damages and cellular senescence in progeria cells. However, the potential role of Sun2 in mechanical stress-induced nuclear damage and its correlation with nuclear decoupling and softening is still not clear. By applying cyclic mechanical stretch to mesenchymal stromal cells (MSCs) of WT and Zmpset24^−/−^ mice (Z24^−/−^, a model for HGPS), we observed much increased nuclear damage in Z24^−/−^ MSCs, which also featured elevated Sun2 expression, RhoA activation, F-actin polymerization and nuclear stiffness, indicating the compromised nuclear decoupling capacity. Suppression of Sun2 with siRNA effectively reduced nuclear/DNA damages caused by mechanical stretch, which was mediated by increased nuclear decoupling and softening, and consequently improved nuclear deformability. Our results reveal that Sun2 is greatly involved in mediating mechanical stress-induced nuclear damage by regulating nuclear mechanical properties, and Sun2 suppression can be a novel therapeutic target for treating progeria aging or aging-related diseases.

## Introduction

Mechanical properties of cells or cytoskeleton change profoundly during aging process or in aging-related diseases [1–4], but the potential changes in the mechanical properties of cell nucleus and related regulatory mechanism remains much less understood. Nuclear envelope (NE) and lamina network localized beneath the nuclear envelope are the key determinants of the mechanical properties of nucleus [5, 6]. However, the mechanism regulating the changes in mechanical properties of nucleus during aging process are still unclear. Also, a variety of tissues (i.e., skin, blood vessels, lung, cardiac muscle, skeletal muscle, and central nervous system, et al.) are known to develop obvious change in mechanical properties during aging or in aging-related diseases, generally featuring increased tissue stiffness or matrix rigidity [1, 2, 4, 7–10]. The increased stiffness of aged tissues can be contributed by the changed mechanical properties of both ECM and cells. Meanwhile, the increased ECM stiffness in aged tissues would generate elevated mechanical stress to the cells, requesting cells to adjust to the stiffer microenvironment for proper functioning and protecting nucleus from mechanical stress-induced damages [11, 12]. Mechanical properties of cell and nucleus are known to be constantly adjusted according to the mechanical properties of their surrounding microenvironment, which greatly influences cell phenotype and fate [11, 13, 14]. However, how this adjusting capacity and regulator mechanism are changed in cells of aged tissues is not clear.

The ability of cells to properly transfer mechanical stress from extracellular matrix (ECM) to nucleus is crucial for protecting nucleus and genome from damages of extreme mechanical stress [15–17]. Increased mechanical stress to the nucleus is able to promote cellular senescence at least via two types of mechanisms: (1) extreme mechanical stress acting on the nucleus may cause DNA damages and genome instability, which has long been proposed as a major causal factors of cellular senescence [18–20]; (2) the increased damage to the structure and integrity of nuclear envelope can lead to increased nuclear DNA (ncDNA) releasing into cytosol, which causes excessive activation of innate immune signaling and innate immune-associated cellular senescence [21–24].

Studies have shown that mechanical stress from ECM is transferred from cytoskeleton to the nucleus via the LINC (linker of the nucleoskeleton and cytoskeleton) complex that spans from nuclear envelop to the perinuclear region [25, 26]. The cell nucleus is tightly integrated into the structural network of the cytoplasm through LINC complexes [25, 26]. LINC complex functions as a structural link bridging the connection of cytoskeleton (i.e., F-actin and microtubule) outside of nuclear envelope and nuclear lamina inside of nuclear envelope. The Sad1 and UNC84 Domain Containing proteins (Suns), mainly Sun1 and Sun2, are the key components of LINC complex, and are key mediators that transfer mechanical stresses from cytoskeleton to nucleus [27–30]. LINC complexes that contain Sun2, but not Sun1, was found to promote focal adhesion assembly by activating RhoA, a critical regulator for the assembly of F-actin cytoskeleton [31]. However, currently the potential influence of Sun proteins on nuclear morphology/architecture or intranuclear functions involving DNA protection is not known. Therefore, we hypothesize that the Sun proteins may fulfill a crucial function in transferring mechanical stress to nucleus and is involved in the process of mechanical stress-induced cellular senescence. Nuclear softening and nuclear decoupling are the 2 main protective mechanism of cells to resist against mechanical stress-induced nuclear damage [15, 32], and we hypothesize that modulation of Sun2 expression may potentially change the status of nuclear softening and nuclear decoupling in progeria cells, which is associated with the capacity of cells to resist against mechanical stress-induced nuclear damage and cellular senescence.

Hutchinson-Gilford Progeria Syndrome (HGPS) is premature aging disease caused by the mutation of LMNA (lamin A) gene [33]. As a nucleoskeletal protein at nuclear lamina, Lamin A is essential for mechanical support of the nucleus [26]. Instead of normal lamin A protein, HGPS cells produce high level of progerin, which accumulates at lamina network and causes dramatic changes of the nuclear architecture and characteristics, including thickening of the nuclear lamina, increased nuclear stiffness, increased nuclear blebbing, and impaired deformation capacity of nucleus [26, 34–36]. These abnormal nuclear architecture and characteristics can consequently induce further devastating outcomes to the cells, such as the disrupted anchoring of chromatin on lamina structure, disorganization of chromatin structure, dislocation and impaired function of telomere, clustering of nuclear pores, delayed response in DNA-damage repair, increased DNA damages, and accelerated senescence [26, 34–38].

Zmpste24^−/−^ (Z24^−/−^) mice have been studied as a reliable animal model for HGPS disease [39, 40]. Our previous study of mesenchymal stromal/stem cells (MSCs) from muscle tissues of Z24^−/−^ mice revealed increased F-actin cytoskeleton stiffness and Sun2 expression in Z24^−/−^ MSCs, which is closely associated with increased nuclear stiffness, nuclear blebbing/micronuclei formation, and activation of senescence-associated innate immune signaling [41]. Here, by studying Z24^−/−^ muscles and MSCs, we further investigated the potential changes of Sun2 expression and its correlation with nuclear abnormalities/damages in progeria cells under mechanical stress, and the potential effect and mechanism of Sun2 suppression in regulating mechanical stress-induced nuclear abnormalities (i.e., nuclear blebbing and DNA damage), the status of nuclear softening and decoupling, and innate immune activation-associated cellular senescence.

## Results

### Expression of Sun2 protein is elevated in muscle cells of Z24^−/−^ mice, especially in cells localized around stiffer ECM (extracellular matrix) in the muscle

**Fig. 1:**
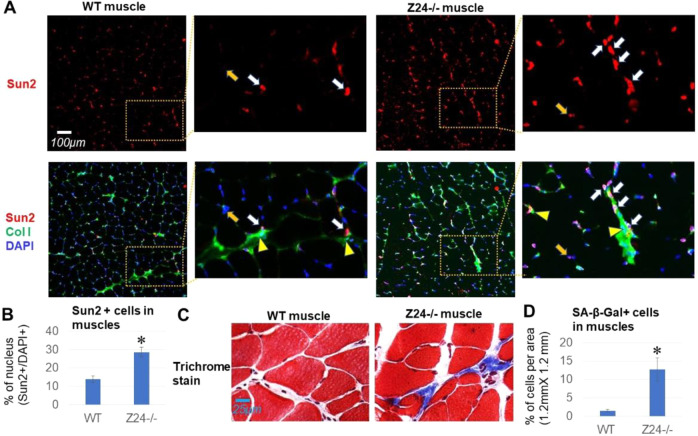
Expression of Sun2 protein is elevated at fibrotic area of muscles in Z24^−/−^ mice. **A** Gastrocnemius (GM) skeletal muscle tissues were harvested from 5-month-old wild-type (WT) and Z24^−/−^ mice. Immunofluorescent staining of Sun2 (red) and Collagen type I (Col I)(green) showed increased number of Sun2-high cells in Z24^−/−^ muscle; also, Sun2-high cells seem to be prone to appear at Collagen type I-rich fibrotic area, which is supposed to have higher mechanical stiffness. White arrows mark the position of Sun2-high cells at Collage type I-rich area, and orange arrows mark the position of Sun2-low cells. Yellow arrow heads mark the area with high collagen deposition (stiffer area). **B** Statistics of the number of Sun2-high cells in muscles. **C** Trichrome staining of WT and Z24^−/−^ muscle tissues to compare the amount of fibrotic tissues. **D** Statistics of the number of SA-β-Gal+ cells in WT and Z24^−/−^ muscles. *N* ≥ 6 for data analysis, with at least three biological replicates and two technical replicates. * indicates *p* < 0.05.

Immunofluorescent staining assay was performed with cryosections of gastrocnemius muscle from aged-matched 5-month old WT and Z24^−/−^ mice. Results showed that, compared to WT muscle, Z24^−/−^ muscles contain a significantly increased number of cells positive with higher Sun2 expression (Fig. 1A, B), and importantly, the cells with higher Sun2 expression (Sun2-high cells) are mostly localized at the area with increased collagen-I deposition (Fig. 1A, B), which is supposed to be fibrotic ECM (extracellular matrix) in aged muscle. It is known that fibrotic ECM or tissue is usually higher in mechanical stiffness than normal ECM or tissues [42], and our observation may indicate that cells at fibrotic area in Z24^−/−^ muscles adjusted to the stiffer mechanical microenvironment of ECM by developing higher Sun2 expression. Consistently, results of trichrome staining also showed that there were increased amount of fibrotic ECM in Z24^−/−^ muscles (Fig. 1C, D).

### Expression of Sun2 protein is increased by stiffer substrate in cell culture

**Fig. 2:**
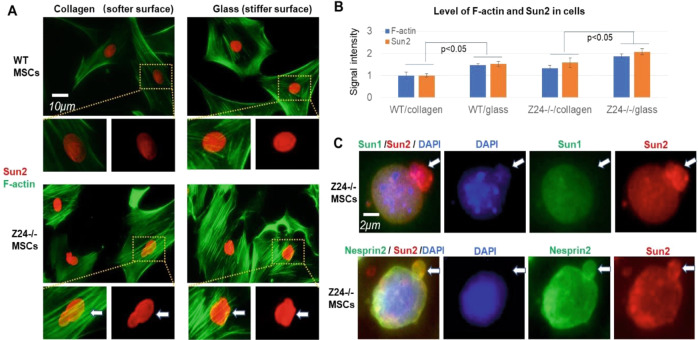
Expression of Sun2 protein and F-actin polymerization are increased in MSCs cultured on stiffer surface, and Sun2 is more enriched in nuclear blebs. **A** Mesenchymal stromal/stem cells (MSCs) were isolated from gastrocnemius (GM) muscle tissues of 5-month-old WT and Z24^−/−^ mice, and cultured on surface with different substrate stiffness (2% collagen-coated plastic, ~50 kPa; non-coated glass, ~50,000 kPa). Immunofluorescent staining of Sun2 (red) and staining of F-actin with Alex Fluo 488 phalloidin (green) showed increased Sun2 expression and F-actin polymerization in WT and Z24^−/−^ MSCs cultured on glass. Arrows mark nucleus with nuclear blebs. **B** Statistics of the level of Sun2 in F-actin in different groups of cells. **C** Immunofluorescent staining of Sun1 and Sun2 in Z24^−/−^ MSCs with nuclear blebs showed the enrichment of Sun2 in nuclear blebs; immunofluorescent staining of Nesprin2 and Sun2 in Z24^−/−^ MSCs with nuclear blebs showed co-localization of Nesprin2 and Sun2 in nuclear blebs. Arrows mark nuclear blebs. *N* ≥ 6 for data analysis, with at least three biological replicates and two technical replicates. * indicates *p* < 0.05.

In order to further verify the potential correlation between Sun2 expression and mechanical properties of ECM substrate observed in muscle tissues above, MSCs from muscles of WT and Z24^−/−^ mice were isolated, and cultured in the plate with varied substrate stiffness. Collagen-coated plastic surface was applied to culture the cells to test the cell response to substrate of low stiffness (2% collagen-coated plastic, ~50 kPa of stiffness), while glass surface was applied to test the cell response to substrate of high stiffness (~50,000 kPa) [43, 44]. Immunofluorescence staining of WT and Z24^−/−^ MSCs showed that, cells cultured on glass surface developed obviously increased Sun2 expression and F-actin polymerization compared to cells culture on collagen-coated surface (Fig. 2A). Meanwhile, the ratio of cells with nuclear blebs in Z24^−/−^ MSCs was increased when being cultured on glass surface (Fig. 2A, B). Immunofluorescence staining of Z24^−/−^ MSCs with nuclear blebs also revealed that, higher level of Sun2 protein, but not Sun1, was specifically present in the nuclear bleb portions (Fig. 2C). Nesprin protein is connected with Sun proteins in LINC complex, and physically links this complex to the actin cytoskeleton in the cytoplasm [45, 46]. We further observed that, both Nesprin2 and Sun2 proteins co-localized in the nuclear bleb portions (Fig. 2C), indicating that LINC complex at the nuclear membrane of nuclear bleb portions contains mainly Sun2 (but not Sun1), and Sun2-containing LINC complex may play a role for the formation of nuclear blebs.

### Expression of Sun2 protein is increased by external mechanical stress

**Fig. 3:**
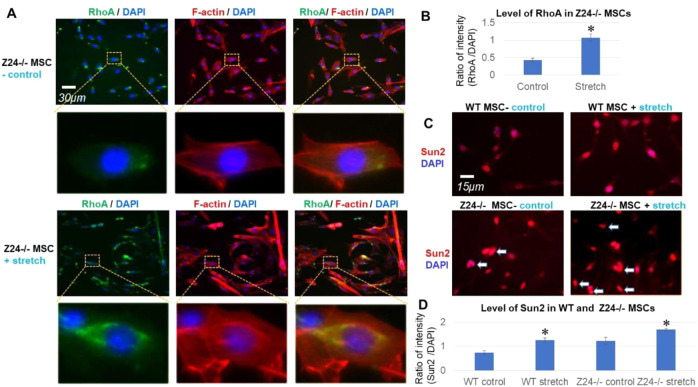
Cyclic mechanical stretch increased RhoA activation, F-actin polymerization and Sun2 expression in Z24^−/−^ MSCs. **A** Z24^−/−^ MSCs were loaded for cyclic mechanical stretch with a FlexCell cell stretching bioreactor (10% uniaxial cyclic stretch at 0.5 Hz of frequency for 24 h). Immunofluorescent staining of RhoA GTPase (green) and staining of F-actin with Alex Fluo 594 phalloidin (red) showed increased level of RhoA protein and F-actin polymerization Z24^−/−^ MSCs after stretch. **B** Statistics of the level of RhoA protein in Z24^−/−^ MSCs with and without stretch. **C** Immunofluorescent staining of Sun2 (red) showed increased Sun2 expression in WT and Z24^−/−^ MSCs after stretch. Arrows mark nucleus with high Sun2 expression or nuclear blebs. **D** Statistics of the level of Sun2 protein in WT and Z24^−/−^ MSC with or without stretch. *N* ≥ 6 for data analysis, with at least three biological replicates and two technical replicates. * indicates *p* < 0.05.

Increased mechanosensitivity was shown to be developed in HGPS cells [47]. As a key component of mechanoresponsive LINC complex, Sun2 is also possibly mechanosensitive. Thus, we examined the potential impact of external mechanical stress on Sun2 expression in Z24^−/−^ MSCs. Extreme mechanical stress was mimicked by applying cyclic mechanical stretch to cells cultured in vitro with a FlexCell cell stretching bioreactor [15, 48], and cells were loaded for cyclic mechanical stretch (10% uniaxial cyclic stretch at 0.5 Hz of frequency) for 24 h. Immunofluorescent staining result showed that, 24 h after stretch, Z24^−/−^ MSCs developed obviously increased activation of RhoA GTPase (a key regulator of F-actin assembly) and F-actin polymerization (Fig. 3A, B), which is a reasonable response of RhoA and actin cytoskeleton to extreme mechanical stress [49] and verifies the reliability of the cyclic mechanical stretch experimental system. Meanwhile, we observed obviously elevated expression of Sun2 protein in Z24^−/−^ MSCs 24 h after stretch (Fig. 3C, D). This result indicates that elevated Sun2 expression may be coupled with increased RhoA activation and cytoskeleton stiffness upon stimulation of extreme mechanical stress in Z24^−/−^ MSCs.

### Suppression of Sun2 expression reduced nuclear blebbing, DNA damage and expression of SASP factors in Z24^−/−^ MSCs treated with cyclic mechanical stretch

**Fig. 4:**
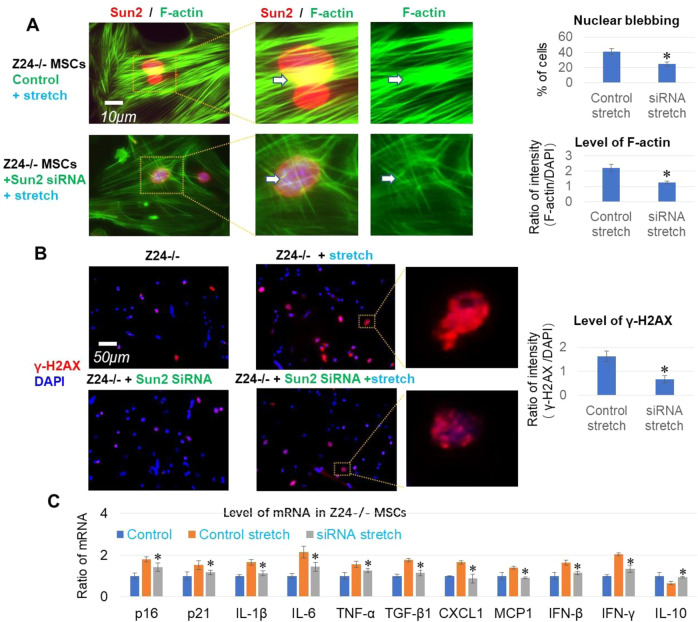
Suppression of Sun2 expression in Z24^−/−^ MSCs decreased F-actin polymerization and nuclear blebbing promoted by cyclic mechanical stretch. **A** Z24^−/−^ MSCs were transfected with silencing RNA (siRNA) for Sun2 or control siRNA for 36 h, and then loaded for cyclic mechanical stretch (10% uniaxial cyclic stretch at 0.5 Hz of frequency for 24 h). Immunofluorescent staining of Sun2 (red) and staining of F-actin with Alex Fluo 488 phalloidin (green) showed decreased level of Sun2, F-actin polymerization and nuclear blebbing in Z24^−/−^ MSCs with Sun2 suppression after stretch. Arrows mark the location of peri-nuclear actin. **B** Immunofluorescent staining of γ-H2Ax (red) (a marker for DNA damage) showed decreased level of γ-H2AX in Z24^−/−^ MSCs with Sun2 suppression after stretch. **C** Real-time PCR assay of senescence markers (p16 and p21), SASP factors (IL1-β, IL6, TNF-α, TGF-β1, MCP1, and CXCL1), effectors of cGAS-Sting innate immune signaling (IFN-β and IFN-γ) and anti-inflammatory factor IL-10 in three groups of Z24^−/−^ MSCs (Z24^−/−^ control, Z24^−/−^ control with stretch, and Z24^−/−^ with Sun2 suppression and stretch). *N* ≥ 6 for data analysis, with at least three biological replicates and two technical replicates. * indicates *p* < 0.05.

Because of the close correlation between elevated Sun2 expression and nuclear blebbing, we continued to examine the potential effect of Sun2 suppression on the formation of nuclear blebs in Z24^−/−^ MSCs. Sun2 expression in Z24^−/−^ MSCs was suppressed by transfection of cells with a specific silencing RNA (SiRNA) for Sun2. Cells were then cultured for 36 h, before being loaded for cyclic mechanical stretch with a FlexCell cell stretching bioreactor for 24 h. Result of immunofluorescent staining revealed that, suppression of Sun2 expression led to a decreased level of nuclear blebbing in Z24^−/−^ MSCs treated with cyclic mechanical stretch (Fig. 4A). This result suggests a beneficial effect of Sun2 suppression in rescuing nucleus of progeria cells from damage of extreme mechanical stress.

Nuclear blebbing or micronuclei formation was shown to be coupled with increased DNA damage and releasing of nuclear DNA (ncDNA) into cytosol, which promotes the activation of innate immune signaling (i.e., cGAS-Sting pathway) and accelerated senescence [22, 23, 50]. Thus, we continued to examine how innate immune-associated cellular senescence was impacted by Sun2 suppression in Z24^−/−^ MSCs treated with cyclic mechanical stretch. Sun2 expression in Z24^−/−^ MSCs was suppressed and cells were loaded for cyclic mechanical stretch. Result of immunofluorescent staining revealed that, suppression of Sun2 expression led to the decreased level of γ-H2AX (an indicator of DNA damage) in Z24^−/−^ MSCs treated with cyclic mechanical stretch (Fig. 4B). Also, the expression of SASP (Senescence-associated secretory patterns) factors, senescence markers (p16 and p21), and effectors of cGAS-Sting innate immune signaling (IFN-β and IFN-γ) was down-regulated in Z24^−/−^ MSCs with Sun2 suppression (Fig. 4C), indicating a delayed progress of innate immune-associated cellular senescence [41].

### Suppression of Sun2 expression promoted nuclear decoupling

We followed to examine the potential cellular mechanism of reduced Sun2 expression in leading to reduced nuclear and DNA damages by extreme mechanical stress. Nuclear decoupling, which is the dissociation of cytoskeleton and nucleus, has been revealed to protect nucleus from mechanical stress-induced damage [32]. Sun proteins bridges the connection between cytoskeleton and nuclear lamina, and therefore the level of Sun2 is possibly directly associated with the degree or strength of this connection. Alexa Fluor 488 Phalloidin staining of F-actin revealed that F-actin polymerization in Z24^−/−^ MSCs with stretch was reduced with Sun2 suppression (Fig. 4A), including F-actin at perinuclear location that is closely associated with changes of nuclear shape [51, 52]. This result suggests that the reduced F-actin polymerization may be part of the cellular mechanism for nuclear decoupling caused by Sun2 suppression in progeria cells.

### Sun2 suppression promotes nuclear decoupling by reducing RhoA activation

**Fig. 5:**
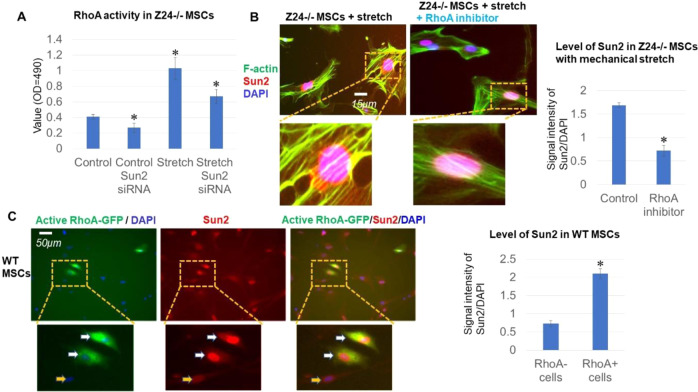
Sun2 suppression in Z24^−/−^ MSCs lead to reduced RhoA activity and Sun2 expression is closely regulated by RhoA activity. **A** RhoA activity was analyzed in Z24^−/−^ MSCs, Z24^−/−^ MSCs with Sun2 suppression, stretched Z24^−/−^ MSCs, and stretched Z24^−/−^ MSCs with Sun2 suppression, with a RhoA G-LISA Activation Assay kit. **B** Inhibition of RhoA activity was performed with a specific RhoA/ROCK inhibitor Y27632 in Z24^−/−^ MSC with stretch, and the level of Sun2 expression, nuclear blebbing, and density of perinuclear F-actin were observed; Statistics of Sun2 level in Z24^−/−^ MSCs with or without Y27632 (10 μM) during mechanical stretch. **C** Activation of RhoA was performed by transfection of WT MSC with a vector overexpressing constitutively active RhoA-GFP, and the level of Sun2 and RhoA-GFP was observed. Arrows: cells with both positive RhoA-GFP signaling and high Sun2 expression. *N* ≥ 6 for data analysis, with at least three biological replicates and two technical replicates. * indicates *p* < 0.05.

We continued to examine the potential molecular mechanism of Sun2 suppression in promoting nuclear decupling. RhoA is the key regulator of F-actin cytoskeleton, and excessive activation of RhoA/ROCK signaling was observed in Z24^−/−^ MSCs [41]. RhoA activity was compared among Z24^−/−^ MSCs, Z24^−/−^ MSCs with Sun2 suppression, stretched Z24^−/−^ MSCs, and stretched Z24^−/−^ MSCs with Sun2 suppression, with a RhoA G-LISA Activation Assay kit. Results showed that, RhoA activity was significantly increased in Z24^−/−^ MSCs with stretch, and Sun2 suppression efficiently reduced RhoA activity in Z24^−/−^ MSCs with or without stretch (Fig. 5A). This result can therefore explain the reduced level of F-actin polymerization in Z24^−/−^ MSCs that was observed in the result of Fig. 4A. These results suggest that Sun2 suppression may have promoted nuclear decoupling by reducing RhoA activation.

### Modulation of RhoA activity in Z24^−/−^ MSCs effectively changed the expression of Sun2

In order to further verify the involvement of RhoA signaling as part of mechanism of Sun2 suppression in reducing mechanical stress-induced nuclear damage, RhoA activity was modulated in Z24^−/−^ MSCs to examine the potential changes in Sun2 expression. First, inhibition of RhoA activity was performed with a specific RhoA/ROCK inhibitor Y27632 in Z24^−/−^ MSC with stretch, and it showed that RhoA inhibition effectively reduced the level of Sun2 expression and nuclear blebbing, as well as the density of perinuclear F-actin (Fig. 5B). Second, consistent activation of RhoA in WT MSC was performed by transfection of cells with a vector overexpressing constitutively active RhoA-GFP, and it showed that cells positive active RhoA-GFP signaling were also positive with higher Sun2 expression (Fig. 5C).

### Suppression of Sun2 expression promoted nuclear softening

**Fig. 6:**
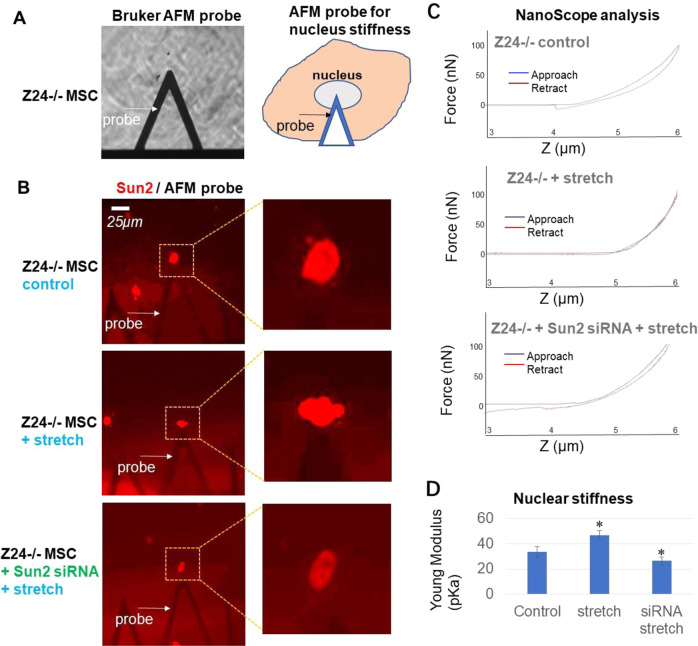
Suppression of Sun2 expression in Z24^−/−^ MSCs lowered nuclear stiffness promoted by cyclic mechanical stretch. **A** Testing the nuclear stiffness of Z24^−/−^ MSCs by atomic force microscope (AFM) loaded with a Bruker AFM probe. **B** Z24^−/−^ MSCs were transfected with Sun2 siRNA or control siRNA for 36 h, and then loaded for cyclic mechanical stretch (10% uniaxial cyclic stretch at 0.5 Hz of frequency for 24 h). Mechanical stiffness at the nucleus was then detected with AFM probe (arrows). Sun2 staining of the cells was performed to localize and identify the nucleus of Z24^−/−^ MSCs in different groups. **C** Mechanical force detected by NanoScope analysis when AFM probe approach and retract from cell nucleus, reflecting the stiffness of nuclear matrix AFM probe traveled through. **D** The nuclear stiffness (kPa) calculated by Young Modulus force. *N* ≥ 6 for data analysis, with at least three biological replicates and two technical replicates. * indicates *p* < 0.05.

We followed to examine whether nuclear softening may also be part of the cellular mechanism for reduced Sun2 expression in leading to decreased level of nuclear and DNA damages by extreme mechanical stress, since increased nuclear stiffness was shown to be developed in HGPS cells [47] and our previous study also revealed increased nuclear stiffness in Z24^−/−^ MSCs [41]. Here we examined whether extreme mechanical stress may change nuclear stiffness of Z24^−/−^ MSCs, and whether Sun2 suppression may reverse this change. Nuclear stiffness was tested by the Atomic force microscopy (AFM) system using a Bruker AFM probe (Fig. 6A, B). The nuclear stiffness of Z24^−/−^ MSCs, which was calculated as Young Modulus force, was significantly elevated after mechanical stretch, and Sun2 suppression in Z24^−/−^ MSCs was shown to effectively reduced the elevated nuclear stiffness promoted by cyclic mechanical stretch (Fig. 6C, D).

### Suppression of Sun2 expression improves nuclear deformability of Z24^−/−^ MSCs

**Fig. 7:**
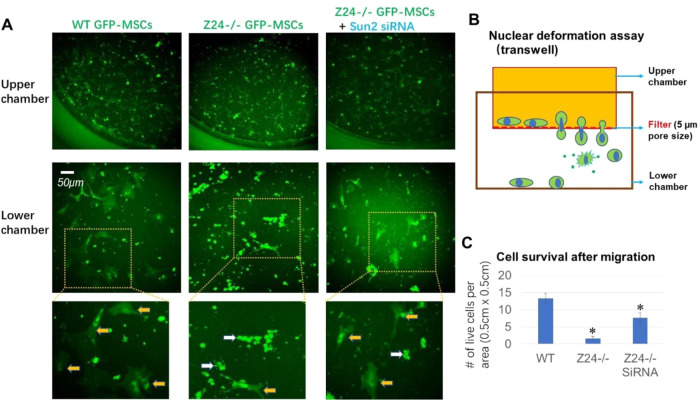
Suppression of Sun2 expression improves the nuclear deformability of Z24^−/−^ MSCs. **A** MSCs (WT MSCs, Z24^−/−^ MSCs, and Z24^−/−^ MSCs with Sun2 suppression) were labeled by expressing GFP tracker and loaded to a transwell cell migration system with the filter of 5 µm in pore size, which forces nucleus to deform dramatically when MSCs migrate through the transwell filter. Live cells that survived the nuclear deformation process and attached on the surface of lower chamber were observed by tracking GFP+ signal 48 h after being seeded on the filter of upper chamber of transwell system. Orange arrows mark live GFP+ cells, and white arrows mark the debris of dead cells. **B** Schematic representation of constricted migration and nuclear deformation assay using transwell. **C** Statistics of the number of live cells in three groups of cells after migration. *N* ≥ 6 for data analysis, with at least three biological replicates and two technical replicates. * indicates *p* < 0.05.

Accumulation of progerin at nuclear envelope of HGPS cells leads to the dramatic changes of the nuclear characteristics, including thickening of the nuclear lamina, increased nucleus stiffness and nuclear blebbing, and impaired nuclear deformability [26, 34–36, 47]. Nuclear deformability is required for cells to properly respond to mechanical forces, cytoskeletal organization and dynamics, cell polarization, and cell migration [25, 34, 51]. Nuclear compression generated by nuclear deformation can lead to increased rupture of nuclear membrane, DNA damage, and apoptosis [16, 52]. Interestingly, Sun2 was found to be potentially involved in regulating nuclear deformation of normal cells [53]. Here we further examined whether Sun2 suppression in Z24^−/−^ MSCs was able to rescue impaired nuclear deformability of progeria cells. MSCs were labeled by expressing GFP tracker and loaded to a transwell cell migration system with the filter of 5 µm in pore size, which is smaller than the regular size of the nucleus and forces nucleus to deform dramatically to migrate through the narrow matrix space of the transwell filter [54]. Cells living on the plastic surface of lower chamber were observed by tracking GFP+ signal 48 h after being seeded on the filter of upper chamber of transwell system. Result showed that, compared to WT MSCs, Z24^−/−^ MSCs could hardly survive after migrating through the filter of transwell (Fig. 7), indicating increased level of nuclear damage and apoptosis caused by the impaired nuclear deformability of Z24^−/−^ MSCs. However, suppression of Sun2 was effective in improving the survival of Z24^−/−^ MSCs after migrating through the narrow matrix space of the transwell filter (Fig. 7), indicating a potentially crucial role of Sun2 protein in regulating nuclear deformability in progeria cells.

## Discussion

Cell nucleus is tightly integrated into the structural network of cytoskeleton in cytoplasm through the LINC complexes on nuclear envelope, which is essential for a broad range of cellular functions, including nuclear positioning and movement in the cells. *LMNA* mutation in HGPS disease causes increased nuclear stiffness and nuclear abnormalities in the progeria cells [26, 34–36, 38, 47, 55]; however, the potential regulatory mechanism of nuclear abnormalities in response to mechanical stress remained unclear.

**Fig. 8:**
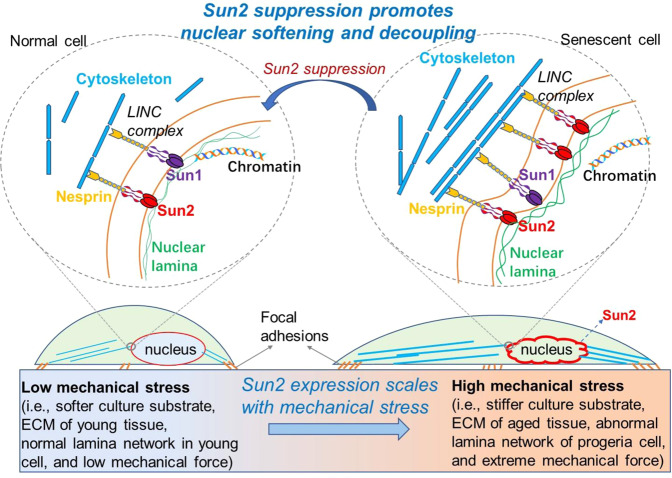
Model for the potential role and mechanism of Sun2 in mediating response of nucleus to mechanical stress in young and senescent cells. The Linker of nucleoskeleton to cytoskeleton (LINC) complex physically connect the nucleus to the cytoplasm. The nuclear lamina is directly connected to Sun1 and Sun2 on the inner nuclear membrane. Sun proteins penetrate through the intermembrane space and bind nesprins on the outer nuclear membrane, which is connected with cytoskeleton. In the situation of lower mechanical stress, both Sun1 and Sun2 are expressed at a relatively lower level in nucleus; however, in the situation of higher mechanical stress, Sun2 expression is specifically elevated. High Sun2 expression can be coupled with increased actin cytoskeleton assembly, RhoA activation, nuclear stiffness and nuclear blebbing, and accelerated cellular senescence. Our results also demonstrated that suppression of Sun2 in progeria cells is able to promote nuclear decoupling, nucleus softening and reduce mechanical stress-induced nuclear damages, and therefore delay the process of cellular senescence.

Our previous study of HGPS disease revealed the crucial role of elevated Sun2 expression in response to cytoskeleton stiffness, nuclear blebbing, micronuclei formation, and changes in RhoA/ROCK activity in progeria cells [41], however, it was unclear whether Sun2 plays a role in mediating mechanical stress-induced nuclear damage, and whether suppression of Sun2 expression may help preventing mechanical stress-induced nuclear damage. Here, by studying progeria cells from Z24^−/−^ mice, we observed that external mechanical stress results in the elevated Sun2 expression i, which is coupled with increased nuclear abnormalities in progeria cells; while the suppression of over-activated Sun2 expression in progeria cells was able to reduce nuclear blebbing and micronuclei formation. Based on these observations, a model for the role of Sun2 in nucleus response to mechanical stress in young and senescent cells is presented as Fig. 8.

Sun proteins (i.e., Sun1 and Sun2) at nuclear envelope are key mediators that transduce mechanical stresses from the ECM and cytoskeleton into the nucleus [27–30]. LINC complexes that contain Sun2, but not Sun1, was found to promote focal adhesion assembly by activating RhoA [31]. Previous observations have shown the role of Sun1 in responding to progerin accumulation in nuclear lamina [27–30]. Our current result, however, has been the first to verify the crucial role of Sun2 (but not Sun1) in mediating mechanical-stress induced nuclear damage and cellular senescence.

Our results showed that the suppression of Sun2 in Z24^−/−^ cells generally resulted in decreased F-actin polymerization, nuclear blebbing, DNA damage, loss of heterochromatin, telomere dislocation, and subsequently innate immune-associated cellular senescence. As a key factor of LINC complex, Sun2 protein interacts closely with actin cytoskeleton in the cytoplasm and nuclear lamina in the nucleus, which determines the degree of mechanical force that can be transferred through cytoskeleton to nucleus [31]. Therefore, the over-activated Sun2 expression in progeria cells may indicate a close and strong coupling between cytoskeleton and nuclear lamina, and thus the extreme mechanical stress was able to exert greater impact on nuclear architecture and characteristics. While, the suppression of Sun2 expression in progeria cells may have reduced the strong coupling between cytoskeleton and nuclear lamina, which avoids or reduces the harmful impact of extreme mechanical stress on nucleus. Thus, it seems that the nuclear decoupling function, which is the decoupling of cytoskeleton and nucleoskeleton and is important for protecting nucleus form mechanical stress-induced damage [32], is greatly compromised in progeria cells, and suppression of Sun2 expression is able to improve the nuclear decoupling function of the progeria cells.

Our previous study of progeria cells also revealed the loss of H3K9me3 protein (a histone modification associated with heterochromatin) from the nucleus [41], indicating a potential important role of heterochromatin in regulating nucleus abnormalities. Interestingly, it was also reported that nuclear softening can be achieved by changes in the level of heterochromatin, and can protect DNA against mechanical stress-induced damage [15]. Therefore, heterochromatin could possibly be involved in the mechanistic explanation of Sun2 function in regulating mechanical stress-induced nuclear and DNA damage.

Elevated level of progerin deposition in nuclear lamina is the direct consequence of LMNA mutation in progeria cell, which was already known to promote the increased stiffness and abnormalities of nucleus [34]. In our previous study of Zmpste24−/− and HGPS cells [41], it was observed higher level of Sun2 is closely associated with increased stiffness and abnormalities of nucleus. Therefore, elevated Sun2 can be a downstream consequence of abnormal nuclear shape caused by Zmpste24 loss in progeria cells. Also, we observed that the expression level of Sun2, but not Sun1 or overall level of Lamin A/C, was significantly changed in progeria cells in contrast to WT cells. Here we further verified that Sun2 is critical for mediating increased nuclear abnormalities and mechanical stress-induced nuclear damages in progeria cells, and the suppression of Sun2 expression is effective in reducing the nuclear abnormalities and damages. Therefore, in addition to increased deposition of prelamin A, the elevated level of Sun2 can be another critical contributor to promote the increased nuclear abnormalities and DNA damages in progeria cells.

In summary, our current results revealed that elevated Sun2 expression is greatly involved in mediating mechanical stress-induced nuclear damage in progeria cells, and suppression of Sun2 expression is effective in reducing mechanical stress-induced nuclear damages, which can be a novel therapeutic strategy for treatment of progeria aging or aging associated diseases.

## Methods and materials

### Animal models

*Zmpste24*^*−/−*^ (*Z24*^*−/−*^*)* mice (B6.129SZmpste24tm1Sgy/Mmucd) were studied as an established model for HGPS. The aged-matched littermates (*Zmpste24*+*/+*) mice born from same *Zmpste24*+*/−* parents were used as wild-type (WT) controls. Both male and female mice were used for this study since both genders are susceptible to HGPS disease. All mice were housed and maintained in the Center for Laboratory Animal Medicine and Care (CLAMC) at UTHealth in accordance with established guidelines and protocols approved by the UTHealth Animal Welfare Committee. Both male and female mice were included in this study.

### Cell isolation and culturing

Muscle-derived Mesenchymal stem/stromal cells (MSCs) were isolated from the gastrocnemius skeletal muscle of WT and Z24^−/−^ mice (~5-month old, male and female) based on their quick adhering capacity to collagen-coated surface/substrate [41], and then sorted with FACS (fluorescence activated cell sorting) according to the positive expression of PDGFR-α. Cells were cultured in DMEM supplemented with 10% fetal bovine serum (FBS).

### Measurement of cellular senescence

The senescent cells in skeletal muscle tissues were identified using the senescence-associated β-Galactosidase (SA-β-gal) Staining Kit (Cell Signaling Technology, Danvers, MA, USA) following the manufacturer’s protocol. The number of cells positive for β-gal activity at pH 6, a known characteristic of senescent cells, was determined.

### Cyclic mechanical stretch assay

Cells were seeded on collagen-coated 6-well BioFlex plates (Flexcell International Corporation) at a density of 30,000–50,000 cells per well. Cells were left to adhere overnight, and then stretched using a Flexcell FX-5000 Tension unit for 24 h with 10% uniaxial cyclic stretch at a frequency of 0.5 Hz.

### RhoA activity assay

Relative RhoA activity was measured in WT MSCs, *Z24*−/− MSCs and Sun2 siRNA-treated *Z24*−/− MSCs with ow without stretch to verify the activation state of RhoA, with a RhoA G-LISA Activation Assay kit (Cytoskeleton Inc.), according to the manufacturer’s instructions. Briefly, cells were lysed and snap-frozen in liquid nitrogen. Approximately 30–40 μg total protein was used for each sample. The active GTP-bound form of RhoA was detected with a specific anti-RhoA antibody. Absorbance readings were obtained by measuring optical density (OD) at 490 nm.

### RhoA inhibition assay

Z24^−/−^ MSCs were treated with RhoA/ROCK inhibitor Y-27632 (EMD Millipore, Billerica, MA, USA) (10 µM) in culture medium during the process of cyclic mechanical stretch, and the potential changes of F-actin polymerization, Sun2 expression, and nuclear blebbing were observed.

### Constant activation of RhoA

Expression of constitutively active RhoA-GFP was performed with amplified plasmid of pcDNA3-EGFP-RhoA-Q63L (Addgene, plasmid #12968). Lipofectamine 3000 (Thermo Fisher) was applied to facilitate the plasmid transfection into WT MSCs.

### Suppression of Sun2 expression with siRNA

WT or Z24^−/−^ MSCs cultured in plates were transfected with a specific siRNA of Sun2 gene (Sun2 SiRNA/mouse from Thermo Fisher; i.e., sense sequence: GCAUCACCAAGACUCGGAATT; anti-sense sequence: UUCCGAGUCUUGGUGAUGCTC) to suppress Sun2 expression, with the assistance of lipofectamine 3000 (Thermo Fisher). Cells transfected with a control SiRNA served as control cells.

### Atomic force microscopy (AFM) testing of nuclear stiffness

Cells were fixed 4% paraformaldehyde and the nuclear stiffness was measured with AFM system at room temperature [56], as described in our study before [41]. The force curves measurements were performed with a Catalyst Bioscope System (Bruker Corporation, Billerica, MA). The AFM was equipped with an inverted light microscope (Olympus IX81) to track the position of cell nucleus and AFM probe. The AFM probe has a material of Non-Conductive silicon nitride, with the spring constant values of ~0.05 N/m (Bruker. Model: MLCT; cantilever: T:0.55 µm). The cantilever sensitivity was calibrated with the NanoScope software by measuring a force curve on a clean silicon wafer. Force curves were acquired at a ramp size of 10 µm, and ramp rate of 1.03 Hz. The Young’s modulus, *E*, was calculated from obtained force curves based on the active curve of “extend” and fit mode of Sneddon (conical) using NanoScope analysis program from the Bruker Corporation. The *F* = 2π*E*1 − *υ*2tan*αδ*2 where *F* = force, *E* = Young’s modulus, *ν* = Poisson’s ratio, *α* = half‐angle of the indenter, and *δ* = indentation depth [56].

### qRT-PCR

Total RNA was obtained from muscle cells or muscle tissues using the RNeasy Mini Kit (Qiagen, Inc., Valencia, CA, USA). Reverse transcription was performed using an iScript cDNA Synthesis Kit (Bio-Rad Laboratories, Inc., Hercules, CA, USA). The sequences of primers were given in Table 1 for SASP factors (IL1-β, IL6, TNF-α, TGF-β1, MCP1, and CXCL1), effectors of cGAS-Sting innate immune signaling (IFN-β and IFN-γ), IL-10 and GAPDH (glyceraldehyde 3-phosphate dehydrogenase). PCR reactions were performed using an iCycler thermal cycler (Bio-Rad Laboratories, Inc.). The cycling parameters used for all primers were as follows: 95 °C for 10 min; PCR, 40 cycles of 30 s at 95 °C for denaturation, 1 min at 54–58 °C for annealing, and 30 s at 72 °C for extension. All data were normalized to the expression of GAPDH.

**Table 1:** Primer sequences.

Gene	Primer sequence
GAPDH	Forward: TCCATGACAACTTTGGCATTG Reverse: TCACGCCACAGCTTTCCA
IL-1β	Forward: GCAACTGTTCCTGAACTCAACT Reverse: ATCTTTTGGGGTCCGTCAACT
TNF-α	Forward: CCTGTAGCCCACGTCGTAG Reverse: GGGAGTAGACAAGGTACAACCC
CXCL1	Forward: CTGGGATTCACCTCAAGAACATC Reverse: CAGGGTCAAGGCAAGCCTC
IL-6	Forward: CTGCAAGAGACTTCCATCCAG Reverse: AGTGGTATAGACAGGTCTGTTGG
MCP1	Forward: TAAAAACCTGGATCGGAACCAAA Reverse: GCATTAGCTTCAGATTTACGGGT
IL-10	Forward: ATTTGAATTCCCTGGGTGAGAAG Reverse: CACAGGGGAGAAATCGATGACA
IFN-β	Forward: CAGCTCCAAGAAAGGACGAAC Reverse: GGCAGTGTAACTCTTCTGCAT
IFN-γ	Forward: ATGAACGCTACACACTGCATC Reverse: CCATCCTTTTGCCAGTTCCTC
TGF-β1	Forward: CTCCCGTGGCTTCTAGTGC Reverse: GCCTTAGTTTGGACAGGATCTG
p16	Forward: CGCAGGTTCTTGGTCACTGT Reverse: TGTTCACGAAAGCCAGAGCG
p21	Forward: CCTGGTGATGTCCGACCTG Reverse: CCATGAGCGCATCGCAATC

### Histology and immunofluorescent staining

Frozen tissue sections were fixed with 10% formalin and cultured cells were fixed with 4% paraformaldehyde. All of the primary antibodies—Lamin A/C (Santa Cruz, Santa Cruz, CA, USA), Sun1 (Novus Biologicals), Sun2 (Abcam), Nesprin 2 (Abcam), γ-H2AX (Cell Signaling), and RhoA (Santa Cruz)—were used at a 1:100 to 1:300 dilution. All slides were analyzed via fluorescence microscopy (Nikon Instruments Inc. Melville, NY) and photographed at ×4–40 magnification. F-actin was stained with Alexa Fluor 488 Phalloidin or Alexa Fluor 594 Phalloidin (Thermo Fisher). The cell nuclei were stained with DAPI. Fibrosis formation in muscle tissues was visualized by Masson trichrome staining with the Trichrome Stain (Masson) Kit (Sigma-Aldrich). Sections were incubated in Weigert’s iron hematoxylin working solution for 10 min, and rinsed under running water for 10 min. Slides were transferred to Biebrich scarlet-acid fuchsin solution for 15 min before incubation in aniline blue solution for another 5 min. Slides were then rinsed, dehydrated, and mounted as earlier. The ratio of the area of fibrotic collagen (blue) to the area of normal muscle (red) was quantified to measure fibrosis formation.

### Cell migration and nuclear deformability assay

WT and Z24^−/−^ MSCs were labeled for GFP expression with the BacMam GFP Transduction Control (BacMam 2.0) (Thermo Fisher), as instructed by manufacturer’s protocol. Cells were loaded to a transwell system with the filter of 5 µm in pore size (Corning Life Sciences, Corning, NY, USA), which is smaller than the regular size of the nucleus and forces nucleus to deform dramatically to migrate through the narrow matrix space of the transwell filter [54]. The surface of filter in the upper chamber were precoated with matrigel (BD Biosciences, San Jose, CA, USA), and DMEM medium containing 15% FBS was added into the lower chamber. In total, 48 h after cells being seeded into the upper chamber of transwell system, live cells attached on the surface of lower chamber were observed by tracking GFP+ signal.

### Measurements of results and statistical analysis

Image analysis was performed using Nikon NIS*-*Elements (Nikon Instruments Inc.) and ImageJ software (version 1.32j; National Institutes of Health, Bethesda, MD). Data from at least six groups of samples were pooled for statistical analysis, with at least three biological replicates and two technical replicates. Prism software (GraphPad) was used to plot graphs and the results were given as the mean ± standard deviation (SD). The statistical significance of any difference was calculated using Student’s *t* test or one-way Anova test. *p* values < 0.05 were considered statistically significant.

## Supplementary information

agreement from new authors

## Data Availability

The data that support the findings of this study are available from the corresponding author upon reasonable request.
