# Supplementary material for: Nuclear softening mediated by Sun2 suppression delays mechanical stress-induced cellular senescence
Source: Cell Death Discov. 2023 May 17;9:167. doi: 10.1038/s41420-023-01467-1 (PMC10192198; doi:10.1038/s41420-023-01467-1)
Supplement: Supplementary file 1 — agreement from new authors [file 41420_2023_1467_MOESM1_ESM.pdf]

Agreement from **Qi Feng:**

https://mail.sdfmu.edu.cn/co...

主　题:	Re:Agreement to be listed as co-author
发件人:	"药学1902冯琦" <viola527@163.com> 2023-5-4 9:57:01
收件人:	"牟晓东" <muxiaodong@sdfmu.edu.cn>

Dear Dr. Mu,
Thank you for your mail. I agree to be listed as co-author.

Best wishes,
Qi Feng

在 2023-05-04 09:42:22，"牟晓东" <muxiaodong@sdfmu.edu.cn> 写道：

> Dear Ms Qi Feng,
>
> We appreciate your contribution to the revision of our manuscript entitled with "Nuclear softening mediated by Sun2 suppression delays mechanical stress-induced cellular senescence", which is being re-submitted to the journal Cell Death & Discovery. Here we are informing you that we need to list you as co-author, and hope to get your agreement.
>
> Best wishes,
> Xiaodong

Agreement from **Ziyue Wang：：**

https://mail.sdfmu.edu.cn/coremail/public/mailprint.j...

主　题:	Agreement to be listed as co-author
发件人:	"_,`≡ˊ、_." <2350972668@qq.com>　　2023-5-4 10:20:35
收件人:	"牟晓东" <muxiaodong@sdfmu.edu.cn>

Dear Dr. Mu， I woud be happy to be co-author .Thank you, teacher, for your help and love, and remember it in my heart. I will strive to improve my academic abilities. Best wishes， Ziyue Wang

---原始邮件---

发件人：牟晓东"

发送时间："undefined"

收件人："2350972668"<2350972668@qq.com>

主题：Agreement to be listed as co-author

Dear Ms Ziyue Wang,

We appreciate your contribution to the revision of our manuscript entitled with “Nuclear softening mediated by Sun2 suppression delays mechanical stress-induced cellular senescence”, which is being re-submitted to the journal Cell Death & Discovery. Here we are informing you that we need to list you as co-author, and hope to get your agreement.

Best wishes,

Agreement from **Ying Li：：**

https://mail.sdfmu.edu.cn/coremail/public/mailprint.jsp?mid...

主　题:	Re:Agreement to be listed as co-author
发件人:	aying092 <aying092@163.com> 2023-5-4 9:55:54
收件人:	"牟晓东" <muxiaodong@sdfmu.edu.cn>

Dear Dr. Mu:

I appreciate your listing me as a co-authour， I agree to be listed as a co-author of the manuscript entitled with "Nuclear softening mediated by Sun2 suppression delays mechanical stress-induced cellular senescence".

Best wishes,

Ying Li

At 2023-05-04 09:40:28, "牟晓东" <muxiaodong@sdfmu.edu.cn> wrote:

> Dear Dr. Ying Li,
>
> We appreciate your contribution to the revision of our manuscript entitled with "Nuclear softening mediated by Sun2 suppression delays mechanical stress-induced cellular senescence", which is being re-submitted to the journal Cell Death & Discovery. Here we are informing you that we need to list you as co-author, and hope to get your agreement.
>
> Best wishes,
>
> Xiaodong

Agreement from **Liwei Shao：：**

主　题:	Re:Agreement to be listed as co-author
发件人:	"邵利伟" <liweishao86@163.com> 2023-5-4 9:55:07
收件人:	"牟晓东" <muxiaodong@sdfmu.edu.cn>

Dear Prof Mu,

I fully agree with your decision. Thanks very much.

Best wishes.

Yours, Liwei Shao

At 2023-05-04 09:42:59, "牟晓东" <muxiaodong@sdfmu.edu.cn> wrote:

> Dear Dr. Liwei Shao,
>
> We appreciate your contribution to the revision of our manuscript entitled with “Nuclear softening mediated by Sun2 suppression delays mechanical stress-induced cellular senescence”, which is being re-submitted to the journal Cell Death & Discovery. Here we are informing you that we need to list you as co-author, and hope to get your agreement.
>
> Best wishes,
